# Supplementary material for: Defect Surface Engineering of Hollow NiCo2S4 Nanoprisms towards Performance-Enhanced Non-Enzymatic Glucose Oxidation
Source: Biosensors (Basel). 2022 Oct 4;12(10):823. doi: 10.3390/bios12100823 (PMC9599600; doi:10.3390/bios12100823)
Supplement: Supplementary file 1 [file biosensors-12-00823-s001.zip › biosensors-1929099-supplementary.pdf]

# Defect Surface Engineering of Hollow $NiCo_2S_4$ Nanoprisms towards Performance-Enhanced Non-Enzymatic Glucose Oxidation

**Xiaomin Lang [1,†], Dandan Chu [1,†], Yan Wang [1], Danhua Ge [1,*] and Xiaojun Chen [1,2,*]**

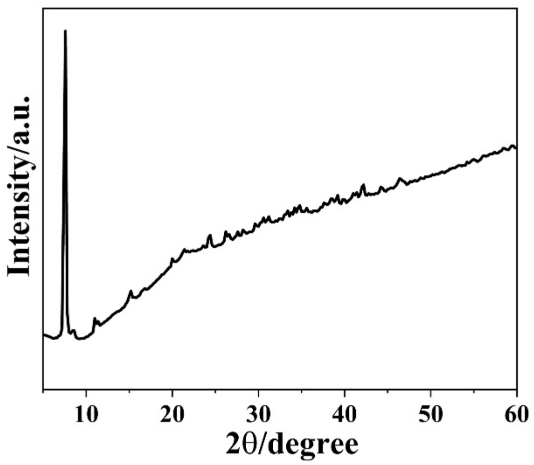


**Figure S1.** XRD pattern of Co/Ni precursors.

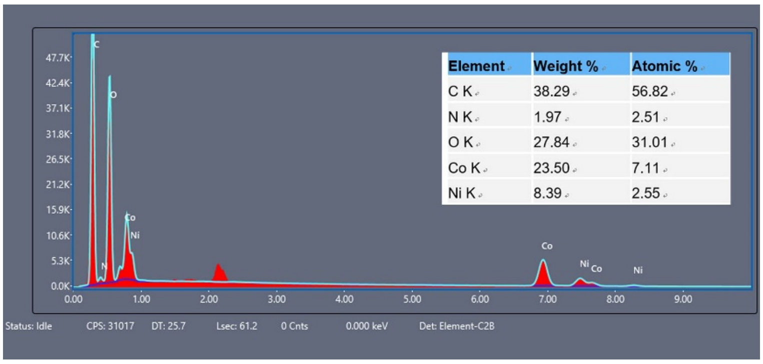


**Figure S2.** EDS spectrum of Co/Ni precursors.

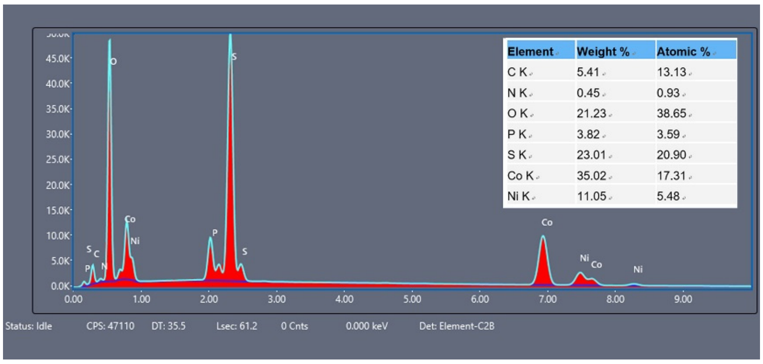


**Figure S3.** EDS spectrum of the P-$NiCo_2S_4$ HNPs.

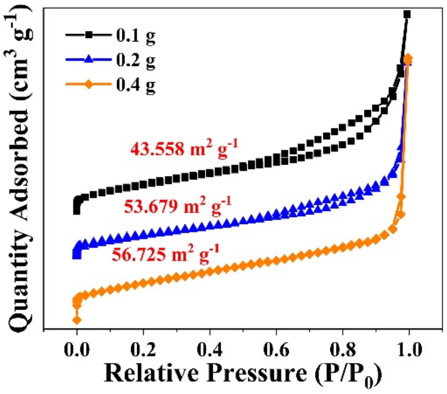


**Figure S4.** $N_2$ adsorption/desorption isotherms of P-$NiCo_2S_4$/ITO with different amounts of $NaH_2PO_2$.

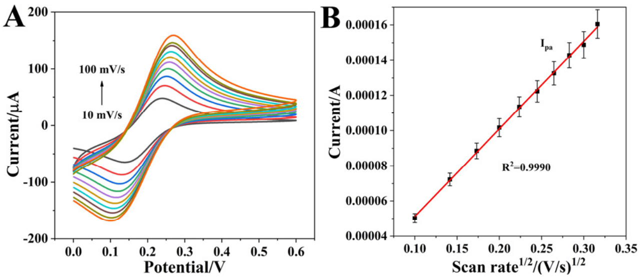


**Figure S5.** (A) CVs of P-$NiCo_2S_4$/ITO in 5 mM $K_3Fe(CN)_6$ solution containing 0.1 M KCl at different scan rates. (B) The linear plot of $I_p$ vs. $v^{1/2}$ of P-$NiCo_2S_4$/ITO.

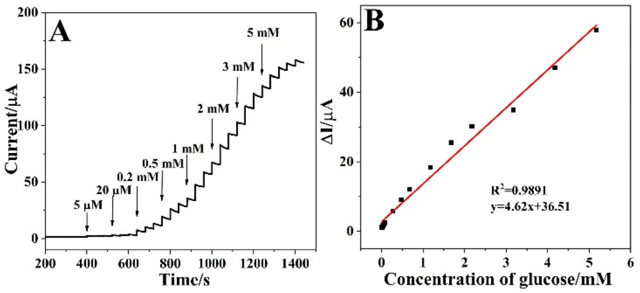


**Figure S6.** (A) Amperometric response of P-$NiCo_2S_4$/ITO with successive addition of glucose at 0.4 V in 0.2 M NaOH in healthy human serum samples. (B) The corresponding calibration curve of the glucose sensor.
